# Opioid Prescriptions at Hospital Discharge Are Associated With More Postdischarge Healthcare Utilization

**DOI:** 10.1161/JAHA.118.010664

**Published:** 2019-01-25

**Authors:** Justin S. Liberman, Lauren R. Samuels, Kathryn Goggins, Sunil Kripalani, Christianne L. Roumie, the VICS (Vanderbilt Inpatient Cohort Study), Justin Bachmann, Susan P. Bell, Katharine M. Donato, Frank E. Harrell, Lindsay S. Mayberry, Amanda S. Mixon, Russell L. Rothman, Jonathan S. Schildcrout, John F. Schnelle, Eduard E. Vasilevskis, Kenneth A. Wallston, Samuel K. Nwosu

**Affiliations:** ^1^ Veterans Health Administration Tennessee Valley VA Health Care System Geriatric Research Education Clinical Center Nashville TN; ^2^ Deparment of Anesthesiology Vanderbilt University Medical Center Nashville TN; ^3^ Department of Biostatistics Vanderbilt University Medical Center Nashville TN; ^4^ Department of Medicine Vanderbilt University Medical Center Nashville TN; ^5^ Center for Health Services Research Vanderbilt University Medical Center Nashville TN; ^6^ Center for Clinical Quality and Implementation Research Vanderbilt University Medical Center Nashville TN

**Keywords:** cardiac disease, heart failure, myocardial infarction, opioid, Heart Failure, Myocardial Infarction, Rehabilitation, Mortality/Survival, Quality and Outcomes

## Abstract

**Background:**

Many patients use opioids for nonmalignant pain, and opioid use in the general population has been associated with poor long‐term outcomes. The use of high‐risk medications, including opioid analgesics, may increase the risk of unplanned healthcare utilization.

**Methods and Results:**

We performed a nested evaluation in the VICS (Vanderbilt Inpatient Cohort Study) (N=3000) on patients with an admitting diagnosis of acute coronary syndrome and/or acute decompensated heart failure. Patient enrollment occurred from October 2011 until December 2015 and involved a single investigational site, Vanderbilt University Medical Center (Nashville, TN). Of the 2495 eligible patients, 501 (20%) were discharged with an opioid prescription and were predominantly white and men, with a median age of 59 (interquartile range, 53–67) years. Our primary outcome was unplanned healthcare utilization, which included emergency department presentation or readmission. Secondary outcomes included mortality and a composite of planned utilization behaviors: cardiac rehabilitation and provider follow‐up within 30 days. Cox proportional hazards models did not show a statistically significant association with increased unplanned utilization (adjusted hazard ratio, 1.06; 95% CI, 0.87–1.28) or mortality (adjusted hazard ratio, 1.08; 95% CI, 0.84–1.39), compared with those without opioids at discharge. Patients discharged with opioids were less likely to complete planned healthcare utilization (adjusted odds ratio, 0.69; 95% CI, 0.52–0.91).

**Conclusions:**

There are decreased odds of planned healthcare utilization among patients with acute coronary syndrome and acute decompensated heart failure discharged with opioid medication. It is imperative to understand how opioid use can affect a patient's relationship with the healthcare system.

**Clinical Perspective**

**What Is New?**

- Patients with acute coronary syndrome and/or acute decompensated heart failure who were discharged with an opioid medication prescription were statistically less likely to complete planned healthcare utilization, defined as a composite of primary care physician follow‐up and participation in cardiac rehabilitation within 30 days of hospital discharge.
- There was a nonsignificant association among patients with acute coronary syndrome and/or acute decompensated heart failure discharged with an opioid medication prescription and the outcomes of mortality and unplanned healthcare utilization, defined as either emergency department visit or hospital readmission.

**What Are the Clinical Implications?**

- In addition to the national focus on opioid‐related overdose and mortality, it is imperative to understand how opioid use can affect a patient's relationship with the healthcare system.
- An acute medical event, such as acute coronary syndrome or acute decompensated heart failure, represents a unique opportunity to reevaluate a patient's medication regimen; reductions in opioid medication prescriptions at discharge may increase primary care physician follow‐up and participation in cardiac rehabilitation.

## Introduction

After hospital discharge, unplanned healthcare utilization (ie, hospital readmission or emergency department use) is both common and costly. Almost 20% of Medicare beneficiaries are readmitted because of any cause within 30 days of their index discharge, with an approximate annual cost to Medicare totaling $17.4 billion.^1^ Certain patients are at the highest risk for unplanned hospital readmissions because of both disease severity and the complexity of the disease process. Patients with acute coronary syndrome (ACS) and acute decompensated heart failure (ADHF) are high‐risk populations who are often readmitted, with 15%^2^ and 23%^3^ 30‐day readmission rates, respectively. Studies focused on multidisciplinary interventions after hospitalization for acute cardiac events have demonstrated reduced hospital readmission in patients with ACS or ADHF if there is early provider follow‐up after discharge.^4^, ^5^, ^6^

The use of high‐risk medications, including opioid analgesics, may increase the risk of unplanned healthcare utilization. Opioid use has become increasingly common in the United States, with sales of prescription opioid analgesics quadrupling from 1999 to 2014.^7^ A study performed by Waljee and colleagues^8^ on patients undergoing elective abdominal surgery found an association between preoperative opioid use and increased length of stay, 30‐day readmission, need for skilled nursing, and higher healthcare costs for up to 1 year after the elective surgical procedure. Increase in hospital readmission in patients with history of prehospital opioid use was also demonstrated by Rogal and colleagues in patients undergoing liver transplantation.^9^

Cardiovascular toxicity related to opioid exposure was demonstrated in an observational cohort of 42 000 patients with a 2.2‐fold increased association with coronary disease in the opioid‐dependent population compared with nonusers.^10^ In a study published by Gomes et al,^11^ opioids were linked to mortality in a dose‐dependent manner in patients with nonmalignant pain. Compared with patients receiving <20 mg morphine daily, mortality was increased, with an odds ratio (OR) of 1.92 (95% CI, 1.30–2.85) for patients with daily morphine doses of 5 to 99 mg and an OR of 2.04 (95% CI, 1.28–3.24) for patients with daily morphine doses of 100 to 199 mg.^11^

Given that opioid use is increasingly common and has been related to mortality and cardiac toxicity, our aim was to test the hypothesis that among patients with ACS or ADHF with a discharge opioid prescription, there would be an increased association with unplanned healthcare utilization and mortality and a decreased association with planned healthcare utilization compared with those discharged without a prescription for opioid analgesics.

## Methods

The data that support the findings of this study are available from the corresponding author on reasonable request.

### Study Design and Overview of Parent Study

This study is a nested evaluation of opioid prescriptions within a prospective cohort of patients with heart disease, the VICS (Vanderbilt Inpatient Cohort Study). The VICS was a prospective cohort study composed of adult patients hospitalized for ACS and/or ADHF. Patient enrollment occurred from October 2011 until December 2015 and involved a single investigational site, Vanderbilt University Medical Center. The purpose of the VICS was to investigate the impact of social and behavioral determinants on postdischarge health outcomes, such as readmission, quality of life, and mortality. The institutional review board of Vanderbilt University Medical Center approved this study, and each patient provided written informed consent and received $30 for study participation. A detailed description of the VICS method has been previously published.^12^

Briefly, VICS patients were recruited if they were 18 years or older and admitted (index hospitalization) with ACS and/or ADHF. Using standardized diagnosis criteria, staff screened patients via electronic medical record review, and a clinician confirmed eligibility. During the index hospitalization, patients completed detailed study questionnaires on social and behavioral risk factors, medication use, and health status. Follow‐up of patients occurred after hospital discharge via telephone interviews at 2 to 3, 30, and 90 days. Patients were retained at a high rate during follow‐up, with 88.0% at 30 days and 86.4% at 90 days after discharge in the parent study. Healthcare utilization for the 90‐day period after discharge was abstracted from charts and included any hospital facility used by the patient. Mortality data were assessed through March 8, 2017 (1.5–4.5 years after discharge).

### Study Sample

Our primary study sample included all patients enrolled in the VICS parent study (N=3000). We then excluded patients who had a hospital stay of <24 hours (eg, brief hospitalization for cardiac catheterization), died during the index hospitalization, underwent coronary artery bypass graft surgery, or used hospice care. We also excluded subjects with incomplete covariates (N=23).

### Exposure: Opioid Prescription at Discharge

We defined the exposure as the presence of an opioid medication on the discharge medication list. All opioid formulations were included in our analysis (Table S1).

For opioids prescribed at discharge, we used the opioid name, dose, and frequency to calculate daily oral morphine equivalents (OMEs) and applied the published morphine equivalent conversion factor specific for each opioid medication (Table S1).^13^ For unscheduled, “as‐needed” opioids, dose was calculated as the maximum dose possible, as prescribed on the discharge medication reconciliation list. In an additional set of analyses, the total daily opioid dose was calculated as the sum of OMEs for both scheduled and as‐needed opioids and categorized into 3 OME groups: <50 mg/d, ≥50 mg/d, or none. Patients who had incomplete opioid information (missing dose, frequency, or type) were excluded from dose analyses.

### Outcomes: Unplanned Healthcare Utilization, Death, and Planned Healthcare Utilization

The primary outcome was time from index hospital discharge until unplanned healthcare utilization within 90 days after discharge. Unplanned utilization was defined as either emergency department visit or hospital readmission. Patients could reach the primary outcome only once and were removed from the risk pool once reaching the outcome. Patients who died within 90 days after discharge without reaching the outcome were censored from the utilization analysis at the time of death.

The 2 secondary outcomes consisted of time to all‐cause mortality and completion of planned healthcare utilization. Time to all‐cause mortality was calculated from index hospital discharge through the end of follow‐up (March 8, 2017). Mortality data were obtained using a combination of data from the Social Security Administration's Death Master File,^14^ follow‐up interview calls, documentation in the electronic health record, family report, and obituaries. During the study, the Death Master File was downloaded monthly and linked to patient records. The importance of using multiple sources to adjudicate vital status has been shown previously in literature and has shown a nearly 100% ascertainment of patient mortality in US‐born individuals.^15^, ^16^, ^17^

Completion of planned healthcare utilization consisted of a composite of physician follow‐up within 30 days of the index hospitalization discharge and, for those referred to cardiac rehabilitation, participation in at least 1 cardiac rehabilitation session within 30 days. Patients who were eligible for cardiac rehabilitation were classified as a failure of planned healthcare utilization if they either missed their rehabilitation session or did not complete provider follow‐up within 30 days. It was assumed that all patients admitted with ACS or ADHF were expected to follow up with a healthcare provider (typically cardiology or primary care) within 30 days after discharge. Patients who died within 30 days of the index hospital discharge were not included in this analysis. Data on unplanned and planned healthcare utilization were obtained via self‐reported telephone calls with the patient or family and verified by chart abstraction. Cardiac rehabilitation centers were contacted directly by a research assistant to validate patient participation among those who had referrals for cardiac rehabilitation.

### Covariates

Study covariates were age, sex (male or female), race (white, black, or other), admission diagnosis (ACS, ADHF, or both), income, socioeconomic status (whether the patient was employed or unemployed; years of education; and income categorized into 5 levels), presence of a regular healthcare provider (yes or no), presence of opioid prescription on admission medication list (yes or no), Elixhauser score,^18^ length of stay for index hospitalization, number of hospital admissions in prior 12 months, and presence of β blocker or aspirin prescriptions at index hospitalization discharge. Discharge disposition (eg, home or skilled nursing facility) was not included because most patients were discharged home after hospitalization.

### Statistical Analysis

Cox proportional hazards models were used to analyze time to unplanned healthcare utilization (within 90 days after discharge) and time to death for patients with opioids on the discharge medication reconciliation list versus patients with no opioids (referent), adjusting for the covariates listed above. The examination of log‐log plots suggested that the proportional hazards assumption was reasonable for both models. To estimate the odds of completing planned healthcare utilization (within 30 days after discharge) by opioid exposure group, we used a logistic regression model adjusting for the same covariates. Adjusted hazard ratios (aHRs) or adjusted ORs and 95% CIs are reported. All analyses were conducted using R (http://www.r-project.org).

### Sensitivity and Subgroup Analyses

Our main analyses relied on direct covariate adjustment to reduce potential confounding. Because the success of this approach depends heavily on the specification of the outcome model, we conducted a sensitivity analysis in a weighted pseudocohort constructed using propensity scores. Cohorts constructed using propensity scores balance the covariates by assigning weights to patients in each group so that the weighted groups closely resemble each other, thus reducing dependence on the specification of the outcome model. In particular, we used propensity score matching weights,^19^ which give the most weight to the patients who most closely resemble those in the opposite group, so that the weighted cohort represents patients who could have conceivably been in either group: discharged with opioid prescription or discharged without opioid prescription. Weighting success in the pseudocohort was evaluated using both a table of patient characteristics and a standardized mean differences plot (Table 1 and Figure S1).

**Table 1:** Patient Characteristics

Patient Characteristics	Discharged WITH Opioids (N=501)	Discharged WITHOUT Opioids (N=1994)
Age, median (IQR), y	59 (53–67)	61 (52–69)
Sex, N (%)		
Male	250 (49.9)	1196 (60.0)
Female	251 (50.1)	798 (40.0)
Race, N (%)		
White	408 (81.4)	1669 (83.7)
Black	85 (17.0)	281 (14.1)
Other	8 (1.6)	44 (2.2)
Diagnosis, N (%)		
Acute coronary syndrome	280 (55.9)	1175 (58.9)
Congestive heart failure	184 (36.7)	696 (34.9)
Both	37 (7.4)	123 (6.2)
Income, N (%)		
<$20 000	138 (27.5)	395 (19.8)
$20 000–$35 000	139 (27.7)	475 (23.8)
$35 000–$75 000	134 (26.7)	578 (29.0)
>$75 000	67 (13.4)	451 (22.6)
Unsure/refused/missing	23 (4.6)	95 (4.8)
Employment status, N (%)		
Employed	89 (17.8)	717 (36.0)
Unemployed/retired	412 (82.2)	1277 (64.0)
Education, median (IQR)		
Highest grade or year completed	13 (12–15)	13 (12–16)
Presence of regular healthcare provider, N (%)	462 (92.2)	1760 (88.3)
Presence of prehospital opioid, N (%)	369 (73.7)	234 (11.7)
Medications at discharge, N (%)		
Aspirin	409 (81.6)	1146 (57.5)
β Blocker	408 (81.4)	1072 (53.8)
Disease severity, median (IQR)		
Elixhauser score*	11 (4–18)	8 (2–16)
Hospital length of stay, d	3 (2–5)	3 (2–5)
Prior healthcare utilization, median (IQR)		
Hospitalizations in 12 months before enrollment	1 (0–3)	1 (0–2)

IQR indicates interquartile range.

* Elixhauser score modified to remove congestive heart failure contribution.

The presence of a prehospitalization opioid without the presence of a discharge opioid may result in drug exposure misclassification because patients with access to opioid medications before their index hospitalization may continue to use these medications after hospitalization even if not prescribed at discharge. To address this concern, we conducted a second sensitivity analysis by redefining our opioid user group to include patients who had an opioid on either their preindex hospitalization medication list or their discharge medication list; we conducted a third sensitivity analysis in which we redefined our opioid user group to include only those patients who had an opioid on their prehospitalization medication list. In addition, because we were concerned about a temporal effect on cohort enrollment, we conducted a sensitivity analysis in which we included patient year of hospital discharge from index hospitalization. Finally, for the 2 outcomes for which death could be considered a competing risk, we conducted sensitivity analyses in which we incorporated death into a composite end point.

We conducted subgroup analyses stratifying by patient age (≥65 or <65 years), sex (male or female), and race (white or nonwhite) to assess associations between opioid prescription and planned healthcare utilization within each of these groups.

### Sensitivity to Unmeasured Confounders

We evaluated the sensitivity of our statistically significant results to the presence of an unmeasured binary confounder. For the outcome of planned healthcare utilization, a potential unmeasured binary confounder, such as frailty, may decrease planned healthcare utilization and may also increase use of opioid medications.^20^, ^21^ We considered hypothetical confounder‐outcome relationships of different strengths and determined the prevalence among opioid and nonopioid users in which our analysis would be inconclusive at the 5% level.

## Results

**Figure 1:**
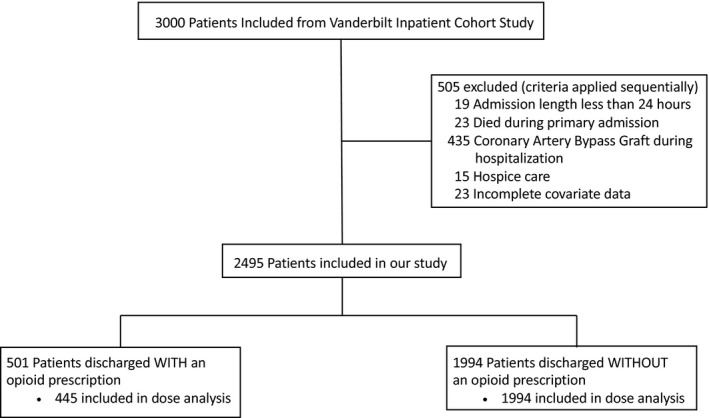
Inclusion and flow of patients in study.

There were 3000 patients enrolled in the VICS cohort. Of these, 505 patients were excluded (length of stay <24 hours, N=19; died during index hospitalization, N=23; coronary artery bypass graft surgery during index hospitalization, N=425; hospice care, N=15; and incomplete covariate data, N=23) for a sample of 2495. There were 501 (20.1%) identified as having a discharge opioid, and 1994 (79.9%) had no discharge opioids (Figure 1). Of the 501 opioid users, 56 were missing elements of the discharge prescription needed to calculate the OME dose and were not included in the dose analysis.

### Patient Characteristics

Patients discharged with opioids were predominantly white (81%), with a median age of 59 (interquartile range [IQR], 53–67) years. Patients discharged without opioid medications were predominantly white (84%) and men (60.0%), with a median age of 61 (IQR, 52–69) years. Admission diagnosis was comparable in both groups (ACS, 56% versus 59%; ADHF, 37% versus 35%; and both ACS and ADHF during the index hospitalization, 7% versus 6%). Patients with opioids at discharge had a higher median Elixhauser comorbidity score (11 [IQR, 4–18] versus 8 [IQR, 2–16]) compared with nonusers. Patients discharged with opioid medication were more likely to have a prehospital prescription for opioid medication (73.7% versus 11.7%, Table 1). On discharge from the index hospitalization, more patients who were discharged with an opioid were also prescribed aspirin (81.6% versus 53.8%) and a β blocker (81.5% versus 53.8%) compared with those not prescribed an opioid. Patients discharged with opioid medication had higher baseline median (IQR) Patient Health Questionnaire scores compared with those not prescribed opioids on discharge (9 [5–13] versus 7 [3–11]).

### Primary Outcome: Time to Unplanned Healthcare Utilization

**Figure 2:**
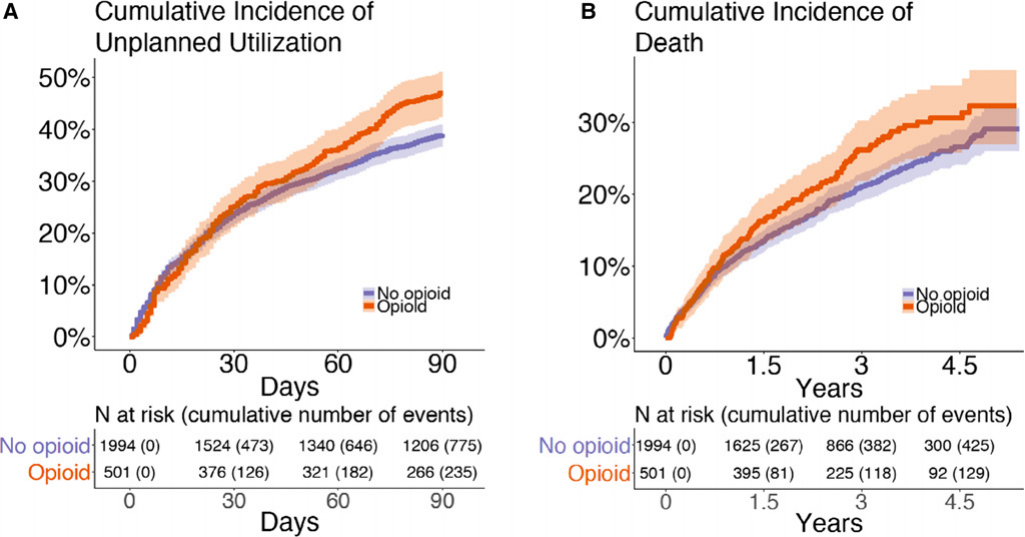
**A**, Cumulative incidence of unplanned healthcare utilization in the 90 days after index hospitalization. **B**, Cumulative incidence of death over 4.5 years of follow‐up after index hospitalization.

**Figure 3:**
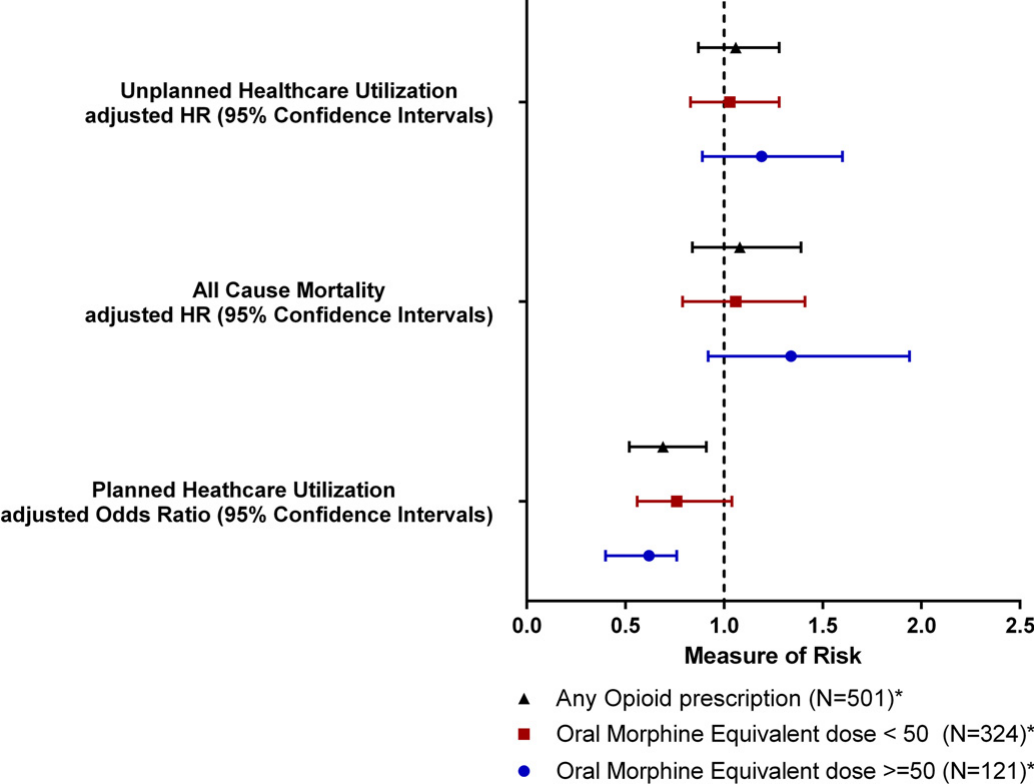
Forest plot of study outcomes (unplanned healthcare utilization, death, or planned healthcare utilization) by discharge oral morphine equivalent dose. *Analysis used any opioid prescription (N=499), oral morphine equivalent dose <50 mg/d (N=323), and oral morphine equivalent dose ≥50 mg/d (N=120). HR indicates hazard ratio.

There were 235 events (71 emergency department visits and 164 hospital readmissions) among patients with a discharge prescription for opioids and 775 events (254 emergency department visits and 521 hospital readmissions) among patients without a postdischarge prescription for opioids. Cox proportional hazards analysis adjusting for covariates yielded an increased point estimate of unplanned utilization for patients discharged with opioids (aHR, 1.06; 95% CI, 0.87–1.28; Table 2 and Figure 2A); however, CIs were wide. The analyses using categorized opioid dose demonstrated that for OME dose <50 mg/d (aHR, 1.03; 95% CI, 0.83–1.28) and OME dose ≥50 mg/d (aHR, 1.19; 95% CI, 0.89–1.60) there remained an increased point estimate of unplanned healthcare utilization compared with nonusers (Table 3 and Figure 3).

**Table 2:** Opioid Prescription at Hospital Discharge and Association With Unplanned Healthcare Utilization, Mortality, and Planned Healthcare Utilization

Variable	Discharged WITH Opioids	Discharge WITHOUT Opioids
Time to unplanned healthcare utilization, N	501	1994
Events, N	235	775
Person‐days	32 072	131 871
Unadjusted rate/1000 person‐days	7.33	5.88
Adjusted hazard ratio (95% CI)*	1.06 (0.87–1.28)	Reference
Death during study period, N	501	1994
Events, N	131	432
Person‐days	512 001	2 043 146
Unadjusted rate/1000 person‐days	0.26	0.21
Adjusted hazard ratio (95% CI)*	1.08 (0.84–1.39)	Reference
Participation in planned healthcare utilization, N	499	1963
Events, N	199	883
Unadjusted rate	0.40	0.45
Adjusted odds ratio (95% CI)*	0.69 (0.52–0.91)	Reference

* Model adjusted for age, sex, race, admission diagnosis, income and socioeconomic status, presence of a regular healthcare provider, presence of prehospitalization opioid prescription, Elixhauser score, length of stay for index hospitalization, number of hospital admissions in prior 12 months, and presence of β blocker or aspirin prescription at index hospitalization discharge.

**Table 3:** Association of Opioid Dose With Risk of Unplanned Healthcare Utilization, Mortality, and Planned Healthcare Utilization

Variable	Unplanned Healthcare Utilization, aHR (95% CI)*	Mortality, aHR (95% CI)*	Planned Healthcare Utilization, aOR (95% CI)†
Discharged WITHOUT opioids	Reference	Reference	Reference
OME dose <50 mg/d	1.03 (0.83–1.28)	1.06 (0.79–1.41)	0.76 (0.56–1.04)
OME dose ≥50 mg/d	1.19 (0.89–1.59)	1.34 (0.92–1.94)	0.62 (0.40–0.97)

aHR indicates adjusted hazard ratio; aOR, adjusted odds ratio; OME, oral morphine equivalent.

* aHR (95% CI) adjusted for age, sex, race, admission diagnosis, income and socioeconomic status, presence of a regular healthcare provider, presence of prehospitalization opioid prescription, Elixhauser score, length of stay for index hospitalization, number of hospital admissions in prior 12 months, and presence of β blocker or aspirin prescription at index hospitalization discharge.

† aOR (95% CI) adjusted for age, sex, race, admission diagnosis, income and socioeconomic status, presence of a regular healthcare provider, presence of prehospitalization opioid prescription, Elixhauser score, length of stay for index hospitalization, number of hospital admissions in prior 12 months, and presence of β blocker or aspirin prescription at index hospitalization discharge.

### Secondary Outcomes: Time to Death and Planned Healthcare Utilization

In the opioid group, 26.1% of patients died, compared with 21.7% in the nonuser group. Cox proportional hazards analysis adjusting for covariates yielded an increased hazard of death for patients discharged with opioids (aHR, 1.08; 95% CI, 0.84–1.39; Table 2 and Figure 2B), but again the results were not significant at the 5% level. Evaluation of the association between categorized opioid dose and time to death demonstrated an aHR of 1.06 (95% CI, 0.79–1.41) for OME dose <50 mg/d and 1.34 (95% CI, 0.92–1.94) for OME dose ≥50 mg/d compared with nonusers (Table 3 and Figure 3).

Among the patients who received opioid prescription at discharge and were still alive 30 days after discharge (N=499), 300 (60.1%) did not complete planned healthcare utilization within 30 days; this included 239 of 499 (47.9%) who did not follow up with a provider within 30 days and 130 of 167 (77.8%) who did not complete any sessions of cardiac rehabilitation. Among nonusers alive at 30 days (N=1963), 1080 (55.0%) did not complete planned utilization. There were 816 of 1963 (41.6%) who did not complete provider follow‐up within 30 days and 481 of 696 (69.1%) who did not complete any sessions of cardiac rehabilitation. Logistic regression adjusting for covariates yielded decreased odds of planned utilization for patients discharged with opioids (adjusted OR, 0.69; 95% CI, 0.52–0.91; Table 2). The analysis of the relationship between categorized opioid dose and planned healthcare utilization demonstrated an adjusted OR of 0.76 (95% CI, 0.56–1.04) for OME doses <50 mg/d and 0.62 (95% CI, 0.40–0.97) for OME doses ≥50 mg/d compared with nonusers (Table 3 and Figure 3).

### Sensitivity and Subgroup Analysis

For the sensitivity analyses conducted using propensity score weighting, the success of the weighting approach in balancing the 2 groups can be seen in the plot of standard mean differences (Figure S1). Results for the weighted analysis were consistent with results from the main analysis, as were the results from the 2 sensitivity analyses in which we reclassified our exposure variable, first to include the presence of preindex and/or postindex hospitalization opioid prescriptions (Table S2) and second to include only prehospitalization prescriptions regardless of postdischarge opioid prescription (Table S3). The results of the sensitivity analyses in which we included patient year of hospital discharge from index hospitalization were also consistent with the results from the main analysis for all outcomes (Table S4).

In our primary analysis (time to unplanned healthcare utilization), death acts as a competing risk. Of 2495 patients, however, only 18 died within the 90‐day window without having first experienced unplanned healthcare utilization. Because this number was so low, we treated these patients as censored in our main analysis. The Aalen‐Johansen plot of the cumulative incidence of unplanned utilization, taking death into account as a competing risk (shown in Figure S2), is nearly identical to Figure 2A, which treats death as censoring. In addition, we conducted a sensitivity analysis in which we used a composite end point of unplanned utilization or death within 90 days. To account for death in our analysis of planned healthcare utilization within 30 days, we conducted a sensitivity analysis in which we included all patients and treated patients who died before completing their planned healthcare utilization as “failures.” The results of both composite end point analyses were close to our original results (Table S5).

Subgroup analyses revealed consistent point estimates for planned healthcare utilization behavior for all subgroups analyzed, with the estimate remaining statistically significant in the 2 largest subgroups, white race and male sex (Table S6 and Figure S3).

### Sensitivity to Unmeasured Confounders

For the planned healthcare utilization outcome, we assessed the sensitivity of our results to a hypothetical unmeasured binary confounder. We found that a confounder with an OR of 0.8 (such that it lowers the odds of completing planned healthcare utilization) would need to be at least 1.7 times as prevalent in the exposed (opioid) group to render the analysis inconclusive at the 5% level. A stronger confounder associated with an OR of 0.6 would need to be at least 1.2 times as prevalent in the exposed group to have the same effect.

## Discussion

Our results extend the current literature on factors that influence postdischarge follow‐up and treatment adherence. This study found a statistically significant association between the presence of opioid prescription at discharge and decreased odds of patients completing outpatient physician follow‐up and cardiac rehabilitation. Both planned healthcare utilization outcomes are evidenced‐based recommendations that reduce the risk of readmission and mortality after an acute cardiac event.

There are many possible mechanisms for these associations. Reduced planned utilization may be inherent to the medication itself; may be caused by lethargy, physical instability, or medication nonadherence^22^; or may be secondary to associated diseases, such as depression.^23^, ^24^ A prospective study of 355 patients with chronic low back pain demonstrated that those with prescriptions equivalent to >50 OMEs per day had 2.7 times the odds of an elevated depressive score relative to patients with low back pain who did not use opioids.^25^ This relationship to depression has been shown to increase patients’ no‐show rates to planned healthcare appointments, increase readmission rates, and decrease adherence to disease self‐management.^26^, ^27^, ^28^, ^29^

Although our primary results were not statistically significant at the 5% level, point estimates were consistent with prior research on opioid use and patient harm.^8^, ^30^, ^31^ We found that patients discharged with opioids had a higher rate of death (26.1% versus 21.7%), and analyses using categorized opioid dose demonstrated results consistent with prior literature on increased OMEs and risk to patients.^11^, ^32^, ^33^

We demonstrated an association with mortality and unplanned healthcare utilization at OME ≥50 mg/d. OME dose ≥50 mg/d was also associated with reduced odds of planned healthcare utilization (adjusted OR, 0.62; 95% CI, 0.40–0.97) compared with patients without opioid prescriptions at discharge. Multiple studies have shown reduced opioid dosing improves pain outcomes and hospital readmission, and our findings further support these studies.^34^, ^35^

Our study does have some limitations. First, we were unable to ascertain the amount of opioid each patient actually ingested. Our assessment of opioids reflects the total daily dose of opioids prescribed to each patient at discharge and does not account for opioids that may have been in the home before the hospitalization. Thus, there is potential for exposure misclassification; however, sensitivity analyses incorporating prehospital opioids into the exposure definition were performed, and results were consistent with those from the main analyses. In addition, our data set did not capture information on opioid prescription duration, which may have influenced likelihood of return for follow‐up care. Second, the use of patient‐reported outcomes could have resulted in outcome misclassification for both unplanned and planned healthcare utilization if patients in 1 group were more or less likely to report rehospitalization or cardiac rehabilitation participation. Approximately 86% of the patients completed the interview series at 90‐day postindex hospital discharge. We supplemented these interviews using objective patient records and have no reason to suspect that outcome reporting was differential by opioid exposure status. Third, there remains the possibility of unmeasured confounding. An unmeasured confounder, such as frailty, could have been associated with both prescription of an opioid and the decreased likelihood of planned healthcare utilization. Our sensitivity analysis exploring the possible impact of unmeasured confounders suggests that our results are robust to at least some degree of imbalance in unmeasured confounders. Fourth, because of changes to the Death Master File, adjudicating vital status was necessary to obtain the most accurate vital status possible; however, it may still underestimate the vital status of patients in our cohort. Finally, the study was performed in a single healthcare system with an 80% white study sample and may not be generalizable to other healthcare settings or populations.

In conclusion, our study demonstrates some of the possible mechanisms by which opioid analgesics impact patients with cardiac disease. Our study identified point estimates consistent with our hypothesis that increased mortality and hospital readmissions occur in cardiac patients prescribed opioid medications; however, we were not able to show statistical significance at the 5% level. Furthermore, we demonstrated a statistically significant finding of decreased odds of planned healthcare utilization among patients admitted with either ACS or ADHF and discharged with opioid medications. This has ramifications on the debate surrounding the current opioid crisis. In addition to the national focus on opioid‐related overdose and mortality, it is imperative to understand how opioid use can affect a patient's relationship with the healthcare system. An acute medical event, such as ACS or ADHF, represents a unique opportunity to reevaluate a patient's medication regimen. Our study supports reductions in opioid prescriptions to improve planned healthcare utilization behaviors. Further research is necessary to understand opioid use and its association with patient self‐care to improve clinical outcomes.

## Appendix

### VICS (Vanderbilt Inpatient Cohort Study) Group

Sunil Kripalani, MD, MSc (principal investigator); Justin Bachmann, MD, MPH; Susan P. Bell, MBBS, MSCI; Katharine M. Donato, PhD; Frank E. Harrell, PhD; Lindsay S. Mayberry, MS, PhD; Amanda S. Mixon, MD, MS, MSPH; Russell L. Rothman, MD, MPP; Jonathan S. Schildcrout, PhD; John F. Schnelle, PhD; Eduard E. Vasilevskis, MD, MPH; and Kenneth A. Wallston, PhD. Senior staff: Courtney Cawthon, MPH; Kathryn Goggins, MPH; and Samuel K. Nwosu, MS.

## Sources of Funding

This study is supported by grant R01 HL109388 from the National Heart, Lung, and Blood Institute, in part by grant 2 UL1 TR000445‐06 from the National Center for Advancing Translational Sciences; and partially supported by the Vanderbilt Department of Anesthesia and the Veteran Affairs (VA) Quality Scholars Fellowship through the VA Office of Academic Affiliations. Its contents are solely the responsibility of the authors and do not necessarily represent official views of the National Institutes of Health or the VA.

## Disclosures

None.

## Supporting information


**Table S1.** Frequency of Post‐Discharge Opioid Prescriptions and Standard Oral Morphine Equivalent Conversion Table
**Table S2.** Results of Exposure Variable Reclassification to Include Pre‐Index Hospitalization Opioid Prescriptions
**Table S3.** Sensitivity Analysis Using Pre‐Hospital Exposure to Opioid Regardless of Post‐Discharge Opioid Prescription
**Table S4.** Results of Models Including Year of Discharge for Index Hospitalization as a Covariate
**Table S5.** Analyses Using Composite Endpoints
**Table S6.** Subgroup Analysis of Intended Healthcare Utilization
**Figure S1.** Absolute standardized mean differences in the original and weighted cohorts.
**Figure S2.** Aalen‐Johansen estimate of cumulative incidence.
**Figure S3.** Subgroup analysis of planned healthcare utilization.
