# Supplementary material for: Opioid Prescriptions at Hospital Discharge Are Associated With More Postdischarge Healthcare Utilization
Source: J Am Heart Assoc. 2019 Jan 25;8(3):e010664. doi: 10.1161/JAHA.118.010664 (PMC6405584; doi:10.1161/JAHA.118.010664)
Supplement: Supplementary file 1 — Table S1. Frequency of Post‐Discharge Opioid Prescriptions and Standard Oral Morphine Equivalent Conversion Table Table S2. Results of Exposure Variable Reclassification to Include Pre‐Index Hospitalization Opioid Prescriptions Table S3. Sensitivity Analysis Using Pre‐Hospital Exposure to Opioid Regardless of Post‐Discharge Opioid Prescription Table S4. Results of Models Including Year of Discharge for Index Hospitalization as a Covariate Table S5. Analyses Using Composite Endpoints Table S6. Subgroup Analysis of Intended Healthcare Utilization Figure S1. Absolute standardized mean differences in the original and weighted cohorts. Figure S2. Aalen‐Johansen estimate of cumulative incidence. Figure S3. Subgroup analysis of planned healthcare utilization. [file JAH3-8-e010664-s001.pdf]

Supplemental Material

Table S1. Frequency of Post-Discharge Opioid Prescriptions and Standard Oral Morphine Equivalent Conversion Table.

Opioid	N (% of total prescriptions)	Conversion Factor
Codeine	1 (0.2)	0.15
Fentanyl patch	11 (1.9)	7.2
Hydrocodone	285 (49.6)	1
Hydromorphone	6 (1.0)	4
Methadone	5 (0.9)	3
Morphine	23 (4.0)	1
Oxycodone	162 (28.2)	1.5
Oxymorphone	6 (1.0)	3
Tapentadol	1 (0.2)	0.4
Tramadol	75 (13.0)	0.1

Table S2. Results of Exposure Variable Reclassification to Include Pre-Index Hospitalization Opioid Prescriptions.

	Presence of Pre-Hospital or Post-Hospital Opioid	Patients WITHOUT Exposure to opioid
Time to Unplanned Healthcare Utilization, N	***735***	***1760***
N events	364	646
Person-Days	44,619	119,325
Unadjusted rate/1000 person-days	8.16	5.41
Adjusted Hazard Ratio (95% confidence intervals)*	1.11 (0.97, 1.27)	Reference
Death During Study Period, N	***735***	***1760***
N events	211	352
Person -Days	729,429	1,825,917
Unadjusted rate/1000 person-days	0.29	0.19
Adjusted Hazard Ratio (95% confidence intervals)*	1.09 (0.91, 1.30)	Reference
Participation with Planned Healthcare Utilization, N	***725***	***1737***
N events	300	782
Unadjusted rate	0.41	0.45
Adjusted Odds Ratio (95% confidence intervals)*	0.85 (0.70, 1.04)	Reference

*Model adjusted for age, sex, race, admission diagnosis, income and socioeconomic status, presence of a regular healthcare provider, presence of prehospitalization opioid prescription, Elixhauser score, length of stay for index hospitalization, number of hospital admissions in prior 12 months, and presence of beta blocker or aspirin prescriptions at index hospitalization discharge

Table S3. Sensitivity analysis using pre-hospital exposure to opioid regardless of post-discharge opioid prescription.

	Pre-hospital exposure to Opioids	NO Pre-Hospital exposure to Opioids
Time to Unplanned Healthcare Utilization, N	***603***	***1892***
N events	305	705
Person-Days	36,170	127,774
Unadjusted rate/1000 person-days	8.43	5.52
Adjusted Hazard Ratio (95% confidence interval)*	1.12 (0.97, 1.28)	Reference
Time to Death, N	***603***	***1892***
N events	184	379
Person -Days	587,168	1,967,980
Unadjusted rate/1000 person-days	0.31	0.19
Adjusted Hazard Ratio (95% confidence interval)*	1.15 (0.95, 1.38)	Reference
Participation in Planned Healthcare Utilization, N	***593***	***1869***
N events	253	829
Unadjusted rate	0.43	0.44
Adjusted Odds Ratio (95% confidence interval)*	0.96 (0.78, 1.19)	Reference

*****Model adjusted for age, sex, race, admission diagnosis, income and socioeconomic status, presence of a regular healthcare provider, presence of prehospitalization opioid prescription, Elixhauser score, length of stay for index hospitalization, number of hospital admissions in prior 12 months, and presence of beta blocker or aspirin prescriptions at index hospitalization discharge

Table S4. Results of models including year of discharge for index hospitalization as a covariate.

	**Discharged WITH Opioids**	**Discharge WITHOUT Opioids**
Time to Unplanned Healthcare Utilization, N	***501***	***1994***
N events	235	775
Person-Days	32,072	131,871
Unadjusted rate/1000 person-days	7.33	5.88
Adjusted Hazard Ratio (95% confidence interval)*	1.06 (0.88, 1.28)	Reference
Time to Death, N	***501***	***1994***
N events	131	432
Person -Days	512,001	2,043,146
Unadjusted rate/1000 person-days	0.26	0.21
Adjusted Hazard Ratio (95% confidence interval)*	1.09 (0.85, 1.41)	Reference
Participation in Planned Healthcare Utilization, N	***499***	***1963***
N events	199	883
Unadjusted rate	0.4	0.45
Adjusted Odds Ratio (95% confidence interval)*	0.68 (0.52, 0.91)	Reference

*Model adjusted for age, sex, race, year of index hospital discharge, admission diagnosis, income and socioeconomic status, presence of a regular healthcare provider, presence of prehospitalization opioid prescription, Elixhauser score, length of stay for index hospitalization, number of hospital admissions in prior 12 months, and presence of beta blocker or aspirin prescriptions at index hospitalization discharge

Table S5. Analyses using composite endpoints.

	Pre-hospital exposure to Opioids	NO Pre-Hospital exposure to Opioids
Time to Unplanned Healthcare Utilization or death within 90 days, N	***501***	***1994***
N events	235	793
Person-Days	32,072	131,871
Unadjusted rate/1000 person-days	7.33	6.01
Adjusted Hazard Ratio (95% confidence interval)*	1.04 (0.86, 1.26)	Reference
Participation in Planned Healthcare Utilization vs. no participation or death within 30 days, N	**501**	**1994**
N events	199	883
Unadjusted rate	0.4	0.44
Adjusted Odds Ratio (95% confidence interval)*	0.71 (0.54, 0.94)	Reference

*Model adjusted for age, sex, race, admission diagnosis, income and socioeconomic status, presence of a regular healthcare provider, presence of prehospitalization opioid prescription, Elixhauser score, length of stay for index hospitalization, number of hospital admissions in prior 12 months, and presence of beta blocker or aspirin prescriptions at index hospitalization discharge

Table S6. Subgroup Analysis of Intended Healthcare Utilization.

		Discharged WITH an Opioid Prescription	Discharged WITHOUT an opioid prescription
**Age >= 65**	Events	122	513
	Patients	327	1222
	Adjusted OR (95%CI)	0.72 (0.51 to 1.03)	Reference
**Age < 65**	Events	77	370
	Patients	172	741
	Adjusted OR (95%CI)	0.64 (0.39 to 1.04)	Reference
**Race: White**	Events	159	732
	Patients	407	1643
	Adjusted OR (95%CI)	**0.69 (0.51 to 0.94)**	Reference
**Race: non-white**	Events	40	151
	Patients	92	320
	Adjusted OR (95%CI)	0.66 (0.33 to 1.32)	Reference
**Sex: Male**	Events	100	523
	Patients	248	1173
	Adjusted OR (95%CI)	**0.63 (0.42 to 0.95)**	Reference
**Sex: Female**	Events	99	360
	Patients	251	790
	Adjusted OR (95%CI)	0.74 (0.50 to 1.11)	Reference

Figure S1. Absolute standardized mean differences in the original and weighted cohorts.

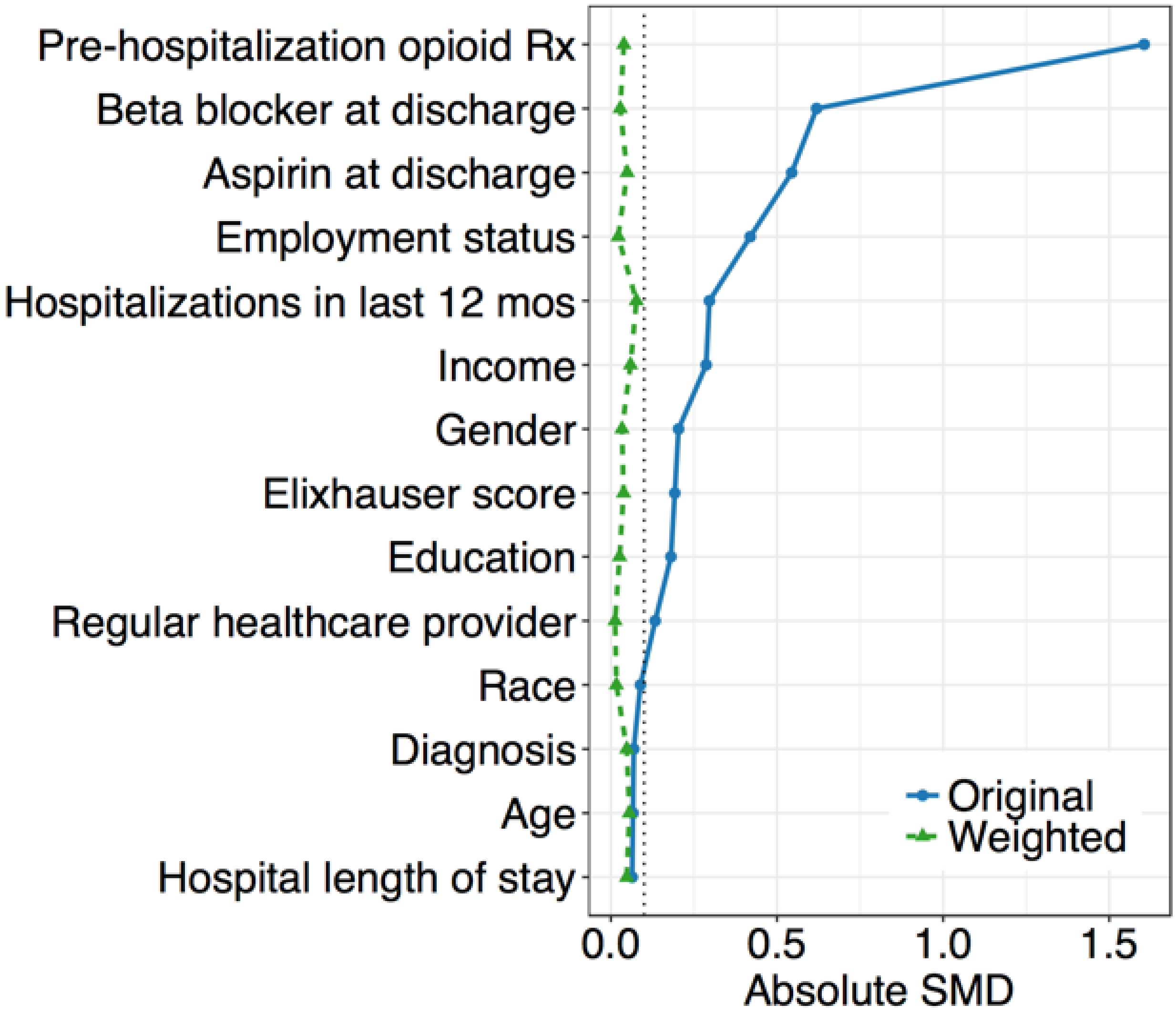

Figure S2. Aalen-Johansen estimate of cumulative incidence.

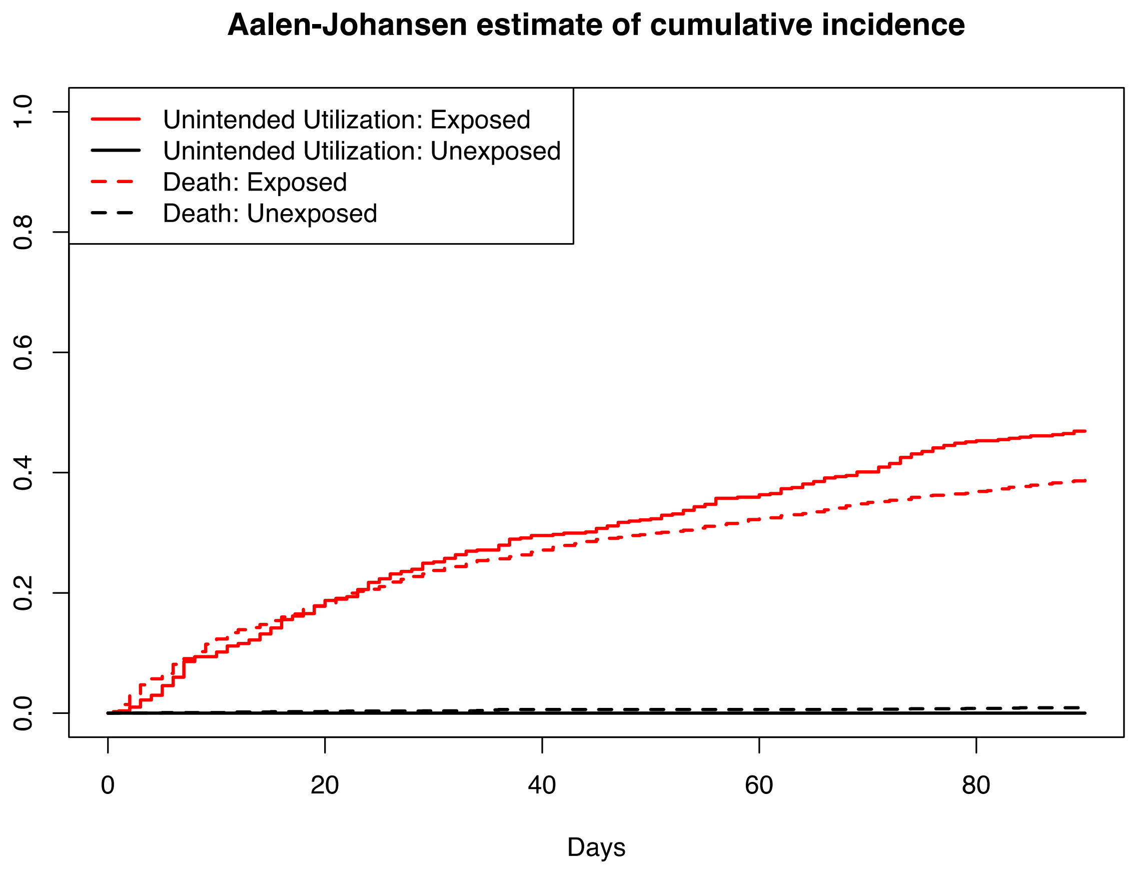

Figure S3. Subgroup Analysis of Planned Healthcare Utilization.

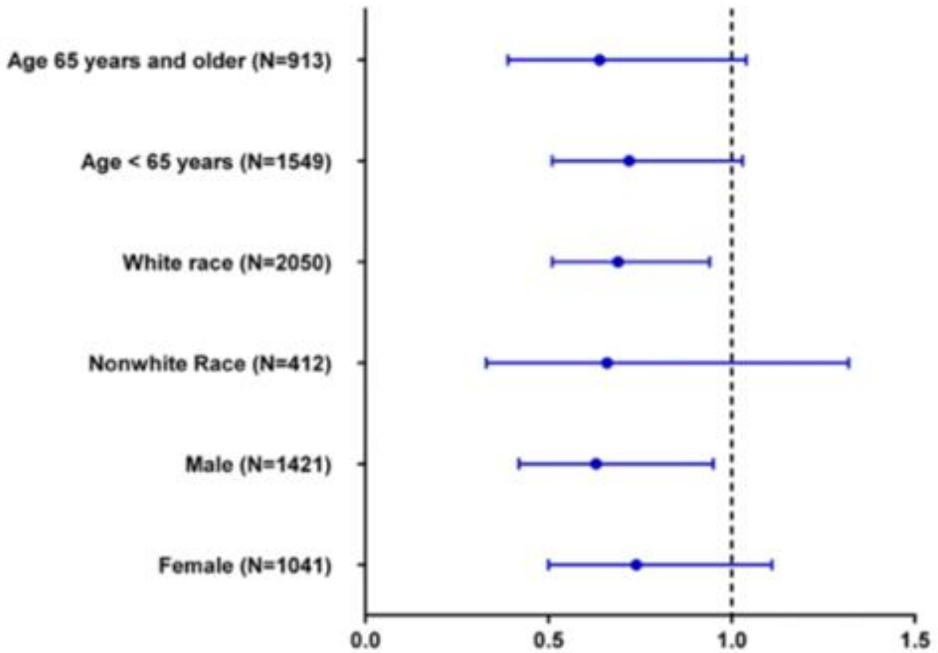

Adjusted Odds Ratio (95% Confidence Intervals)

Planned Healthcare Utilization
